# Particle Size Distribution of Airborne Microorganisms and Pathogens during an Intense African Dust Event in the Eastern Mediterranean

**DOI:** 10.1289/ehp.10684

**Published:** 2007-12-07

**Authors:** Paraskevi N. Polymenakou, Manolis Mandalakis, Euripides G. Stephanou, Anastasios Tselepides

**Affiliations:** 1 Hellenic Centre for Marine Research-Crete, Heraklion, Greece; 2 Environmental Chemical Processes Laboratory, Department of Chemistry, University of Crete, Heraklion, Greece; 3 Department of Maritime Studies, University of Piraeus, Piraeus, Greece

**Keywords:** African dust, bacterial community composition, microbial transport, particle size distribution, pathogens

## Abstract

**Background:**

The distribution of microorganisms, and especially pathogens, over airborne particles of different sizes has been ignored to a large extent, but it could have significant implications regarding the dispersion of these microorganisms across the planet, thus affecting human health.

**Objectives:**

We examined the microbial quality of the aerosols over the eastern Mediterranean region during an African storm to determine the size distribution of microorganisms in the air.

**Methods:**

We used a five-stage cascade impactor for bioaerosol collection in a coastal city on the eastern Mediterranean Sea during a north African dust storm. Bacterial communities associated with aerosol particles of six different size ranges were characterized following molecular culture–independent methods, regardless of the cell culturability (analysis of 16S rRNA genes).

**Results:**

All 16S rDNA clone libraries were diverse, including sequences commonly found in soil and marine ecosystems. Spore-forming bacteria such as Firmicutes dominated large particle sizes (> 3.3 μm), whereas clones affiliated with Actinobacteria (found commonly in soil) and Bacteroidetes (widely distributed in the environment) gradually increased their abundance in aerosol particles of reduced size (< 3.3 μm). A large portion of the clones detected at respiratory particle sizes (< 3.3 μm) were phylogenetic neighbors to human pathogens that have been linked to several diseases.

**Conclusions:**

The presence of aerosolized bacteria in small size particles may have significant implications to human health via intercontinental transportation of pathogens.

In the last decade, the increase of desertification has resulted in a concomitant intensification of atmospheric dust loadings (Moulin and Chiapello 2006). Furthermore, El Niño events have coincided with increased flux of Saharan dust across the Atlantic (Prospero and Lamb 2003). Moulin et al. (1997) have estimated that dust flux from the Saharan-Sahel region to the atmosphere is approximately 1 billion tons/year. In addition to the effect of airborne dust on visibility and Earth’s climate through the processes of atmospheric radiation balance, photochemistry, and cloud formation, these dusts can also exert a direct impact on human health (Taylor 2002). The World Health Organization (WHO) has identified drought and dust storm activity in the sub-Saharan region of Africa as causing regional outbreaks of meningococcal meningitis (in 1996 there were ~ 250,000 cases and 25,000 deaths) (Griffin 2007; WHO 2003).

Recently, dust events have been shown to introduce a significant pulse of microorganisms (Griffin 2007) and other microbiological materials (i.e., cellular fragments, fungal spores) into the atmosphere (Jaenicke 2005). However, to a large extent, the distribution of microorganisms in different particle sizes has been ignored, although it could have significant implications regarding the dispersion of microorganisms around the world. Study of the particle size distribution of microorganisms will enhance our knowledge *a*) of the species of microbes able to be transported over long distances and thus affect remote areas, and *b*) how climate change could increase the health risk from microbial pathogens.

Traditionally, the detection and enumeration of airborne microorganisms has been conducted using light microscopy and/or culture-based methods. However, these analyses are time-consuming and laborious, lack sensitivity and specificity (Stetzenbach et al. 2004), and offer just a glimpse of the biological agents present (< 1% of environmental bacteria can be cultivated) (Amann et al. 1995; Hugenholtz et al. 1998; Pace 1997). The development of various techniques based on community molecular analysis has freed researchers from culturing biases and allowed characterization of community structure [e.g., 16S and 18S ribosomal RNA (rRNA) genes for bacteria and microeukaryotes, respectively] (Amann et al. 1995).

In the present study, we used molecular-based methods to analyze the microbial components of bioaerosol samples collected from the atmosphere of a coastal city (Heraklion, Crete) of the eastern Mediterranean Sea during an intense African dust event. The study area is subject to frequent and severe Saharan dust events (Gerasopoulos et al. 2006). Air sampling was carried out during 24–25 February 2006 when large quantities of dust were exported from northern Africa to southern Europe (NASA 2006). The aim of the study was to investigate the microbial quality of size-distributed aerosol particles during a dust storm by using a high-volume pump equipped with a five-stage cascade impactor for efficient genomic DNA extraction (Radosevich et al. 2002). The composition of the airborne microorganisms was determined by cloning and sequencing the 16S rRNA genes. To the best of our knowledge, this is the first report of size-distributed airborne bacteria during a Saharan storm using molecular-based methods.

## Materials and Methods

### Sample collection and chemical analyses

**Figure 1:**
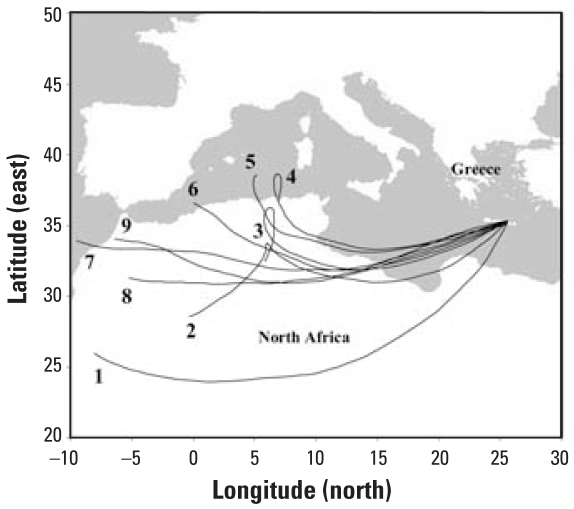
HYSPLIT back-trajectories of air masses arriving at the sampling station during the dust storm over the eastern Mediterranean Sea. Plots show 3-day air mass back trajectories for every 6 hr between 0600 hours EEST (Eastern European Summer Time) on 24 February 2006 and 0000 hours EEST on 26 February 2006 at 3,000 m altitude (Draxler and Rolph 2003). Trajectories: 1, 24 February at 0600 hours; 2, 24 February at 1200 hours; 3, 24 February at 1800 hours; 4, 25 February at 0000 hours; 5, 25 February at 0600 hours; 6, 25 February at 1200 hours; 7, 25 February at 1800 hours; 8, 26 February at 0000 hours; 9, 26 February at 0600 hours.

Air sampling was carried out in a coastal city, Heraklion (25°11′N, 35°19′E), on the eastern Mediterranean Sea during a strong Saharan dust event (1430 hours on 24 February through 1300 hours on 25 February). Heraklion, located on the north coast of Crete, is where most of the population of the island resides. The severe dust event significantly reduced the visibility in the study area. This was further confirmed by HYSPLIT_4 back trajectories (Draxler and Rolph 2003; Figure 1) and satellite images published by NASA (2006).

Size-segregated aerosol samples were collected using a 5-stage Sierra-Anderson high-volume cascade impactor (model 235; Staplex Company, Brooklyn, NY, USA) operated at a constant flow rate of 740 L/min. The sampling system was placed on a 5-m-high platform (located at the University of Crete campus) to reduce possible affects from near-surface sources. Particles were separated into six fractions in the following size ranges: F11, > 7.9 μm; F12, 3.3–7.9 μm; F13, 1.6–3.3 μm; F14, 1–1.6 μm; F15, 0.55–1 μm; and F27, an additional back-up filter that collected particles < 0.55 μm. We used precombusted (420°C) glass fiber filters, and we disinfected all sampler compartments with isopropanol before sampling. After sampling, filters were collected under sterile conditions and stored at −20°C until further analysis. Total organic carbon and nitrogen concentrations were determined using a CHN 2400 analyzer (PerkinElmer, Waltham, MA, USA) (Hedges and Stern 1984). Proteins were extracted in sodium hydroxide, and then neutralized and quantified spectrophotometrically using the ND-1000 spectrophotometer (NanoDrop, Wilmington, DE, USA) calibrated to bovine serum alvumin.

**Figure 2:**
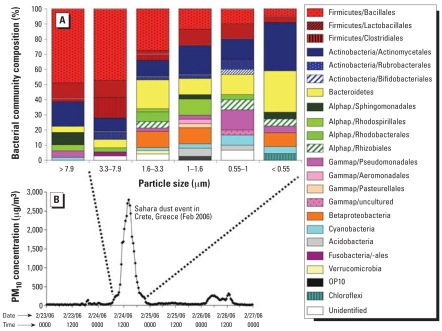
Analysis of dust particles from the Sahara dust event by bacterial community composition and by PM_10_ concentration at different time points. (*A*) Bacterial community composition in particles of different sizes. (*B*) PM_10_ concentrations during the Sahara dust event. Abbreviations: Alphap., Alphaproteobacteria; Gammap, Gammaproteobacteria.

Although the levels of suspended particulate matter during the dust event (up to 2,800 μg/m^3^) were several orders of magnitude higher than the typical background levels at this location (~ 15 μg/m^3^) (Figure 2B), there could be some influence from resuspension of local dust. It is also possible that bare vegetation on the island could serve as source of microbes during the movement of the dust cloud (Prospero et al. 2005). Moreover, some of the detected microbes may also originate from any source that lies along the trajectory of the transported Saharan air masses. A direct verification of Saharan dust as a source of airborne microorganisms would require information regarding microbial populations of the different sources and an extensive comparative analysis. A detailed investigation about the source regions of microbes was out of the scope of the present study; therefore, we did not collect control samples for comparative purposes.

### DNA extraction, cloning, and RFLP (restriction fragment length polymorphism) screening

For each cascade filter, only a small piece was cut (about 12%) and used for total DNA extraction using the UltraClean Soil DNA kit (MOBIO Laboratories, Carlsbad, CA, USA). Bacterial 16S rRNA genes were amplified using polymerase chain reaction (PCR) with the universal bacterial primers 27F (5′-AGRGTTTGATCMTGGCTCAG-3′) (Vergin et al. 1998) and 1492r (5′-GGYTACCTTGTTACGACTT-3′) (Lane 1991). For each sample, 16 replicate PCR reactions of 20 μL were amplified in a PerkinElmer Cycler with initial denaturation at 94°C for 3 min followed by 30 cycles of 1 min at 94°C, 1 min annealing at 50°C, 3 min primer extension at 72°C, and a final extension at 72°C for 7 min. Each tube contained 1–4 ng of target DNA, PCR buffer [10 mM Tris–HCl (pH 9), 50 mM potassium chloride, 0.1% Triton X-100, and 2 mM magnesium chloride], 100 nM of each primer, 200 mM of each deoxyribonucleotide triphosphate, and 0.25 U Taq DNA polymerase (Invitrogen, Carlsbad, CA, USA). All PCR products were pooled and precipitated under vacuum (SpeedVac; Heraeus Instruments, Hanau, Germany) followed by gel purification using the Qiaquick PCR purification kit (Qiagen, Valencia, CA, USA). The concentration of PCR products generated from the different filters was determined spectrophotometrically using the ND-1000 spectrophotometer. For each filter, 10 ng of PCR product was cloned into the pCR 4-TOPO vector and transformed into One shot TOP10 chemically competent cells of *Escherichia coli* using the TOPO TA Cloning kit (version O, Invitrogen) as recommended by the manufacturer.

At least 100 positive clones from each clone library (selected by blue and white screening) were transferred to 96-well plates and incubated overnight at 37°C in Luria–Bertani medium containing kanamycin at 50 mg/mL. Aliquots of the individual clones were archived at −80°C in 7% dimethyl sulfoxide and/or washed by pelletizing cells in a 30-min centrifugation at 10,000 × *g*, followed by supernatant removal by low-speed centrifugation (< 500 rpm) of inverted plates. Pelletized cells were resuspended in 30 μL sterile and ultraviolet (UV)-irradiated Milli-Q ultrapure water (Nanopure, Barnstead, IA, USA). Cells were lysed by heating at 98°C for 10 min, followed by agitation. The lysates were used (1:10 vol/vol) as templates in a PCR amplification of the insert using external (vector) primers M13f-20 (5′-GTAAAACGACGGCCAG-3′) and M13r (5′-CAGGAAACAGCTATGAC-3′; Invitrogen) to avoid co-amplification of *E. coli* host-cell DNA. PCR amplification was carried out for 25 cycles as described above before annealing at 55°C. Positive transformants (clones carrying an insert of correct size) were identified by agarose gel electrophoresis as described above. RFLP analysis was carried out to classify clones into operational taxonomic units. Aliquots (5 μL) of individual PCR products were digested with two four-cutting restriction enzymes (*HhaI* and *HaeIII* ) for 16 hr according to instructions supplied by the manufacturer (Invitrogen). After inactivation of the enzymes (20 min at 85°C), fragments were sized by electrophoresis on a 2% agarose gel (2 hr, 80 V, 10°C). Fragments were recorded using ethidium bromide staining and UV transillumination. We used a 100-bp DNA ladder (Invitrogen) for determination of fragment size. The resulting (RFLP) patterns were then used to classify clones into operational taxonomic units (OTUs).

### Sequencing and phylogenetic analysis

A total of 256 clones from the majority of the detected OTUs were sequenced on an ABI 3700 96-capillary sequencer (Applied Biosystems, Foster City, CA, USA) using the BigDye terminator kit (v.3.1; Applied Biosystems). This generated a high-quality read of between 450 and 780 bases. Using Chimera Check software included in the Ribosomal Database Project II (Michigan State University, East Lansing, MI, USA), we identified eight of the sequences as most likely being chimeras; these were discarded from further analysis. The remaining 248 sequences were compared to GenBank entries using BLAST (Basic Local Alignment Search Tool; National Center for Biotechnology Information, Bethesda, MD, USA) in order to select reference sequences. Sixteen sequences were affiliated with eukaryote organelles, and 11 sequences were not affiliated with any of the known DNA sequences and thus were excluded from further analysis. The remaining 221 clones (49 from F11, 37 from F12, 47 from F13, 37 from F14, 30 from F15, and 21 from F27) were used for phylogenetic analysis. In addition, representative clones (a total of 98) were also sequenced at both directions, resulting in near full-length 16S rDNA sequences of approximately 1,500 bp. The partial 16S rRNA gene sequences, including the closest related sequences determined by BLAST, were imported into ARB software, version 2.5b (Techische Universität München, München, Germany) (Ludwig et al. 2004). All sequences were aligned using the integrated aligner tool and the fast aligner option, followed by manual alignment of the sequences to closely related sequences in the ARB database. We calculated phylogenetic trees by applying the maximum parsimony method. The robustness of tree topologies was confirmed by maximum parsimony analysis (Ludwig et al. 2004) with 100 bootstrap replications (Felsenstein 1981). Values < 50 were removed. The partial 16S rDNA sequences were deposited in GenBank under accession numbers EF682864–EF683084.

### Species richness

We estimated species richness of each clone library using the RFLP-based distribution of clones in different OTUs as previously described (Polymenakou et al. 2005). Species richness was estimated using the nonparametric Chao estimator S*_1_ = *S*_obs_ + (*a*^2^/2*b*), where *S*_obs_ is the number of 16S rDNA clones observed, *a* is the number of clones observed just once, and *b* is the number of clones observed twice (Chao 1984). We estimate the SD using the equation SD = *b*[(*a*/4*b*)^4^ + (*a*/*b*)^3^ + (*a*/2*b*)^2^].

## Results

### Aerosol characteristics

During the Saharan dust storm over the eastern Mediterranean region in February 2006, airborne particulate matter of ≥ 10 μm in aerodynamic diameter (PM_10_) started to rise significantly above the urban level, reaching up to 2,800 μg/m^3^ (Figure 2B). Back trajectories indicated that the air mass during the event arrived in Crete from the northwest African desert (Figure 1). Concentrations of the collected particles ranged from 18.11 μg/m^3^ at filter F11 to 66.05 μg/m^3^ at filter F13. Maximum organic carbon and protein concentrations were recorded at F27 (Table 1).

### Clone libraries coverage

**Figure 3:**
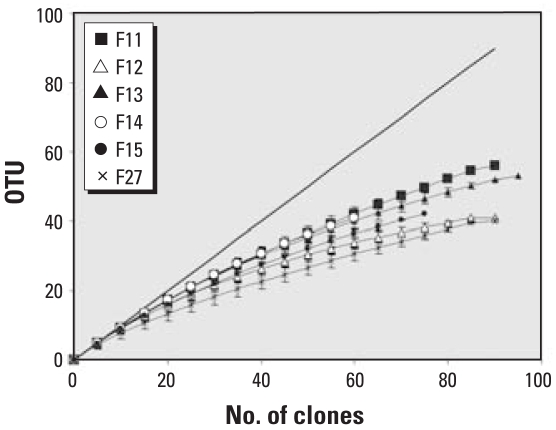
Rarefaction analysis of 16S rDNA sequence heterogeneity in clone libraries from the six particle size ranges collected during the Saharan dust storm in the eastern Mediterranean Sea. The total number of screened clones are plotted against unique OTUs identified by RFLP analysis. Error bars indicate SD; the diagonal line represents the 1:1 relationship where each clone is unique.

The richness and evenness of 16S rDNA-based phylotypes were determined by RFLP analysis of 60–93 clones from each of the six clone libraries (Figure 3). We compared a total of 489 clones. We identified 56 different OTUs among the 88 screened clones from the F11 clone library, whereas we identified 41 of 86, 53 of 93, 41 of 60, 42 of 75, and 40 of 87 from the F12, F13, F14, F15, and F27 clone libraries, respectively. Coverage analysis indicated that the bioaerosol libraries represented approximately 41–70% of the total number of OTUs of the original filters, providing a dependable inventory of the bacterial 16S rRNA gene sequences present in the bioaerosols.

### Taxonomic groups

All 16S rDNA clone libraries were diverse and included sequences commonly found in soil and marine ecosystems. Phylogenetic analysis of the partial rDNA (Figure 2A and Supplemental Material available online at http://www.ehponline.org/members/2007/10684/suppl.pdf) revealed that sequences grouped with the following phyla: Firmicutes, Actinobacteria, Bacteroidetes, Alphaproteobacteria, Gammaproteobacteria, Betaproteobacteria, and Cyanobacteria. Only a few bacterial sequences were affiliated with Acidobacteria, Fusobacteria, Verrucomirobia, Chloroflexi, and the candidate division OP10.

Spore-forming bacteria such as Firmicutes (40.3% of the total sequenced clones) were frequently encountered in all clone libraries, representing 61.2, 70.3, 34.0, 24.3, 20.0, and 9.5% of the clones from filters in order of reduced particle size (Figure 2A). All sequenced clones were grouped in two major classes (*Bacilli*, *Clostridia*) containing cultured representatives from Mali in West Africa, a known source region for dust storms (i.e., GenBank accession numbers AY211114 and AY211153; Kellogg et al. 2004) (Supplemental Material available online at http://www.ehponline.org/members/2007/10684/suppl.pdf).

All sequences affiliated with Actino-bacteria (18.6% of the total sequences) grouped in nine major families (*Micrococcaceae*, *Microbacteriaceae*, *Streptomycetaceae*, *Geodermatophilaceae*, *Microsphaeraceae*, *Propionibacteraceae*, *Nocardioidaceae*, *Bifidobacteraceae,* and *Rubrobacteraceae*), which also contained cultured strains previously isolated from the Mali region (Supplemental Material available online at http://www.ehponline.org/members/2007/10684/suppl.pdf). Four clones were closely related with a bacterium clone obtained from the Red Sea (GenBank accession no. AJ309537).

Of the sequenced clones, 12.2% were affiliated with Bacteroidetes; these were the most abundant at particle sizes < 0.55 μm (Figure 2A). Only one clone was distinct from cultured species: Alphaproteobacteria (10.4% of the total sequenced clones) were unevenly distributed among the libraries (2.7–13.5%), matching previously cultured representatives such as *Sphingomonas*, *Methylobacterium,* and *Paracoccus* species. Clones affiliated with Gammaproteobacteria accounted for 5.4% of the total sequenced clones. The distribution of clones varied largely among the libraries (0–16.7%) that included cultured relatives of pathogenic bacteria (i.e., *Acinetobacter johnsonii*, *Acinetobacter lwoffi*), the psychrophilic bacterium *Psychrobacter phenylpyruvicum,* and ammonia-oxidizing bacteria such as *Nitrosovibrio tenuis*. Betaproteobacteria (4.9%) were encountered only at filters F13, F14, and F27, whereas only 2.7% of the total sequenced clones were affiliated with the phylum Cyanobacteria. The other phyla (i.e., Fusobacteria, Verrucomicrobia, Acidobacteria, OP10) were only minor components (~ 0–5.4%) in each of the six libraries. Only 2.3% of the sequenced clones could not be affiliated with any known bacterial group.

### Pathogenic relatives

The detection of signatures from pathogenic bacteria was evident from the analyses of 16S rRNA genes. However, we did not attempt to search for human pathogens using culture techniques because of the laborious and selective incubation conditions needed for their culture (i.e., a high-level biosafety laboratory, blood agar plates) (Murray et al. 1999).

Approximately 24% of the sequenced clones were closely related to potential human, plant, and animal pathogens (Supplemental Material available online at http://www.ehponline.org/members/2007/10684/suppl.pdf). Almost half of them (43% of the total pathogens) were detected at particle sizes < 3.3 μm. Some of the identified pathogens are of particular interest. *A. lwoffi* (found only with 0.55–1 μm particles; up to 99.2% sequence similarity) and *A. johnsonii* (found with particles > 0.55 μm; up to 99.5% sequence similarity) have been linked to bacteremia and meningitis (Ku et al. 2000). We detected *Haemophilus parainfluenzae* (98.5% sequence similarity) at a particle size range of 1–1.6 μm; *H. parainfluenzae* is responsible for acute bacterial meningitis in infants and young children and for chronic pulmonary disease in adults (Foweraker et al. 1993). *Streptococcus pneumoniae* and S*treptococcus mitis* (up to 99.4% sequence similarity), which cause pneumonia, bacterial sinusitis, acute otitis, and meningitis, were also detected with particles ranging from 0.55 to 7.9 μm in size (Balsalobre et al. 2006; Centers for Disease Control and Prevention 1997). We found *Sphingomonas* species (opportunistic human, animal, and plant pathogens), which are widely distributed in the natural environment and induce several nosocomial infections (Ammendolia et al. 2004), were most abundant in > 7.9 μm particles (up to 98% sequence similarity). Phylogenetic neighbors to *Propionibacterium acnes* (up to 99.7% sequence similarity), suspected to induce pathologic reactions such as endocarditis (Zedtwitz-Liebenstein et al. 2003), were also detected at all particle sizes. One clone obtained from the F14 filter shared 100% sequence similarity with *Streptococcus gordonii* (strain ATCC 10558; American Type Culture Collection, Manassas, VA, USA), an oral pathogen and a significant causative bacterium for infective endocarditis (Kawamura et al. 1995).

## Discussion

Over the past few decades the increase in the amount of African dust flux has been attributed to the ongoing drought in North Africa that began in the 1970s (Prospero and Lamb 2003). Satellite images show that African dust is transported across the Mediterranean Sea to Europe and then crosses the Atlantic Ocean, affecting distant areas. Outbreaks of Saharan dust over the eastern Mediterranean region are very frequent in winter and transitional seasons (October–May) and minimal during the summer (Kubilay et al. 2000). Trajectories indicate that the major source of dust pulses to the eastern Mediterranean originate from northwestern Africa. In a recent study, Kellogg et al. (2004) identified aerosolized microbes using culture techniques from dust events in Mali, West Africa. These cultured microbes represent only a small fraction of what is present in the atmosphere. Contrary to culture techniques, the rRNA sequence approach for microbial identification does not depend on viability under laboratory conditions.

In the present study, we investigated the composition of airborne bacteria in the atmosphere of the eastern Mediterranean during a Saharan dust storm, using air particle size distribution sampling and molecular-based approaches. Despite the extremely high aerosol mass concentration experienced during that event, we cannot exclude the possibility that some of the detected microorganisms could have been derived from sources other than the Saharan region. In a recent study, Prospero et al. (2005) indicated that the concentrations of viable (colony-forming) bacteria and fungi in the atmosphere of the Caribbean were essentially uncorrelated with the levels of transported dust from Africa, implying that Saharan dust was not the sole source of airborne microbes. In our case, the microbial content of Saharan dust could have been mixed with soil particles from land surfaces of northern Africa and Crete along the advection of southerly air masses to the sampling site. Information regarding the microbial populations of soils in these regions is not available; therefore, their contribution to our samples cannot be evaluated.

Clone libraries were constructed for each of the six particle-size ranges. We observed diverse bacterial phylotypes commonly found in soil and marine ecosystems, as well as on human skin. Most of the bacteria types identified were gram positive, accounting for 58% of the total sequenced clones. Most clones were closely related to the bacterial strains that have been detected in Mali (Kellogg et al. 2004). By constructing large clone libraries we were able to identify bacteria that are missed when using culture-dependent methods. Recently, Brodie et al. (2007) used clone libraries and high-density DNA microarrays to detect a diverse bacterial community in the urban aerosols of two large U.S. cities. Using these highly selective techniques, they were able to identify pathogenic members including environmental relatives of bioterrorism significance. However, they did not attempt to investigate the particle size distribution of the detected microorganisms.

Because culture-independent studies of bacteria that can be transported for long distances have yet to be published, the primary aim of the present study was to examine the size distribution of aerosolized bacteria. Phylogenetic analyses revealed that the atmospheric microbial community structure depends on particle size. Spore-forming bacteria, such as Firmicutes, dominated large particle sizes, whereas clones affiliated with Actinobacteria (found commonly in soil) and Bacteroidetes (widely distributed in the environment) gradually increased their abundance in aerosol particles of reduced size.

Up to now, a large number of European and non-European time-series studies have shown an association between human mortality and exposure to small-size particles (Griffin 2007; WHO 2006). Such particles are believed to pose the greatest human health risk by penetrating to the gas-exchange region of the lungs and interfering with lung function (Taylor 2002). It has been estimated that one-half the dust mass consists of particles < 2.5 μm diameter, which possess extremely low deposition velocities (Prospero 1999, 2006). This size range (accumulation mode) has extremely large atmospheric residence time and thus features a high potential for long-range dispersal. But can these dust particles transfer bacterial pathogens for long distances? In the present study, a large fraction of the clones detected at respiratory particle sizes (< 3.3 μm in size) were phylogenetic neighbors to human pathogens that have been linked to several diseases such as pneumonia, meningitis, and bacteremia or suspected to induce pathologic reactions such as endocarditis (i.e., *S. pneumoniae*, *S. mitis*, *S. gordonii*, *H. parainfluenzae*, *A. lwoffi*, *A. johnsonii*, *P. acnes*).

Our study reveals the presence of numerous pathogens at small particle sizes, thus implying their potential for long-range transboundary atmospheric transportation, and foreshadows their negative impact on human health, as well as agricultural and ecosystem health. Thus, further long-term studies on the particle size distribution of aerosolized microbes across the earth in conjunction with viability profiling of the migrating microorganisms will provide a valuable perspective for the fields of environment and public health.

## Figures and Tables

**Table 1:** Environmental characteristics of the bioaerosol samples collected during the Saharan dust storm using the cascade impactor sampler.

Parameter	F11	F12	F13	F14	F15	F27
Particle size (μm)	> 7.9	3.3–7.9	1.6–3.3	1–1.6	0.55–1	< 0.55
Particle concentration (μg/m^3^)	18.11	63.20	66.05	47.79	21.43	39.43
Percent organic carbon	0.40	0.29	0.56	0.86	0	3.92
Percent nitrogen	0.15	0.21	0.33	0.14	0.10	0.32
Carbon/nitrogen ratio	2.67	1.41	1.71	6.26	0	12.10
Percent prot/mass	1.08	0.37	0.48	0.60	0.54	2.05

prot/mass, proteins to particle mass.
